# Antimicrobial stewardship without infectious disease physician

**DOI:** 10.1002/jgf2.172

**Published:** 2018-04-17

**Authors:** Junpei Komagamine

**Affiliations:** ^1^ Department of Internal Medicine National Hospital Organization Tochigi Medical Center Utsunomiya Tochigi Japan

To the Editor,

I read with great interest the recent article reported by Murakami et al.^1^ The authors conducted a before‐and‐after study to investigate the effects of an antimicrobial stewardship team (AST) without an infectious disease physician (IDP) on several outcomes in patients with candidemia. They concluded that non‐IDP AST was associated with improved adherence to guidelines for managing candidemia, but not with improved 30‐day mortality in patients with candidemia. However, I think that important information on the prognosis of the five patients who received no antifungal treatment in the preintervention group is lacking in this article.

If all five patients died, it seems problematic that 30‐day mortality in the intervention group was similar to that in the preintervention group, which included these five patients. If these five patients survived at discharge without antifungal treatment, five (16.7%) of 30 patients with candidemia were considered not to have true candidemia that required treatment. This means that the clinical judgment not to treat for candidemia in these patients was appropriate, although most candida isolated from positive blood cultures reflects true candidemia.^2^ However, given that no gold standard exists for differentiating pathogens from contaminants,^3^ it is interesting that 16.7% of patients with candidemia were managed without any antifungal treatment. Thus, information on the prognosis of these five patients who received no antifungal treatment in the preintervention group is important. Moreover, assessing the clinical significance of candidemia, as in a previous study,^2^ would help to interpret the results of this study. Finally, the initial sentence in the Primary Outcome section of the Results is incorrect. This sentence should read as follows: “Of the intervention group, 11 of 46 patients (23.9%) had died by day 30, compared with 7 of 30 patients (23.3%) in the preintervention group.”

Given that a past study reported improved mortality in patients with candidemia by AST intervention with an IDP,^4^ the IDP's clinical judgment may be the most important aspect of improving the prognosis in patients with candidemia. Therefore, more discussion is needed regarding why the AST without an IDP did not improve mortality despite improving adherence to the guidelines for managing candidemia.

## CONFLICT OF INTEREST

The authors have stated explicitly that there are no conflicts of interest in connection with this article.
